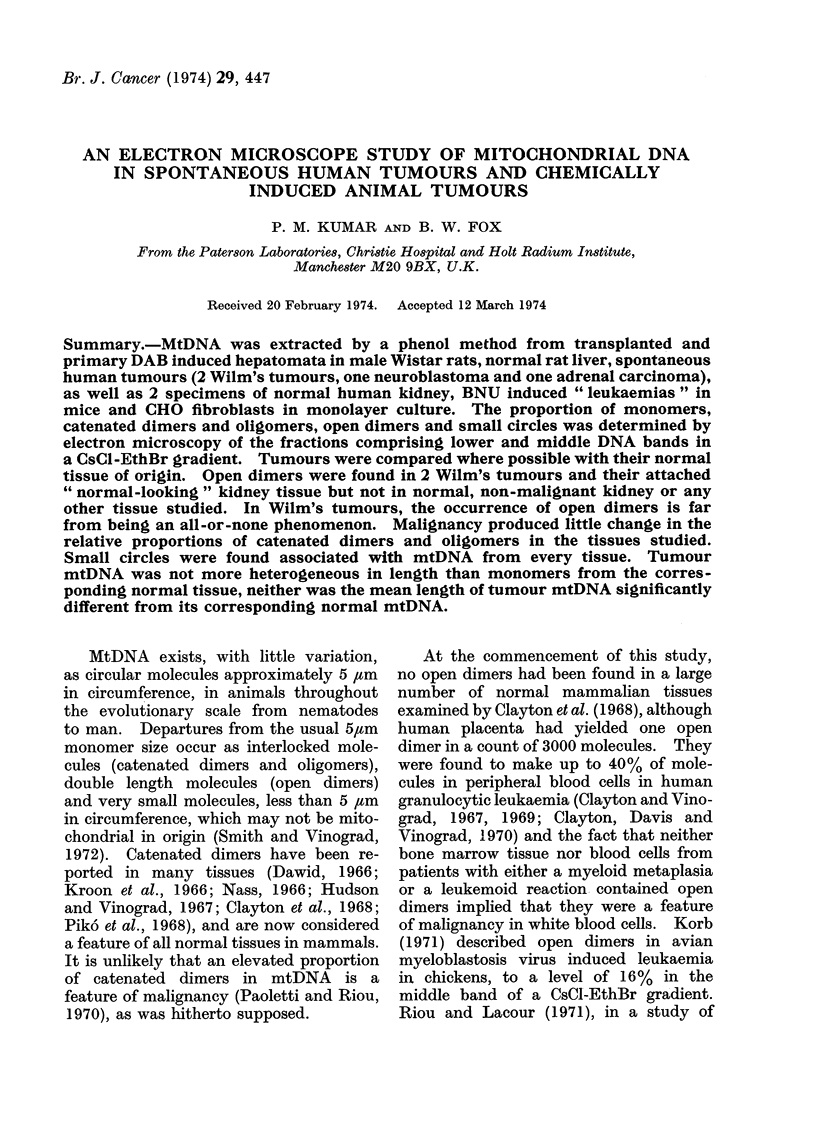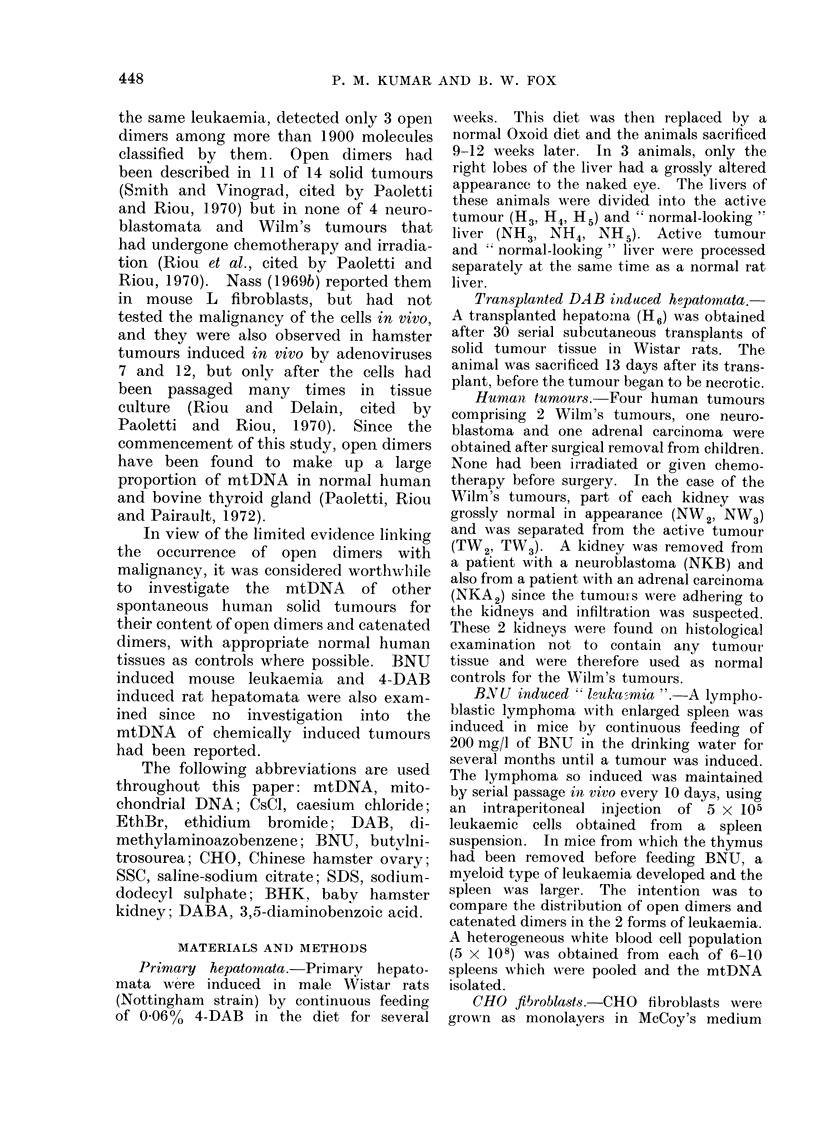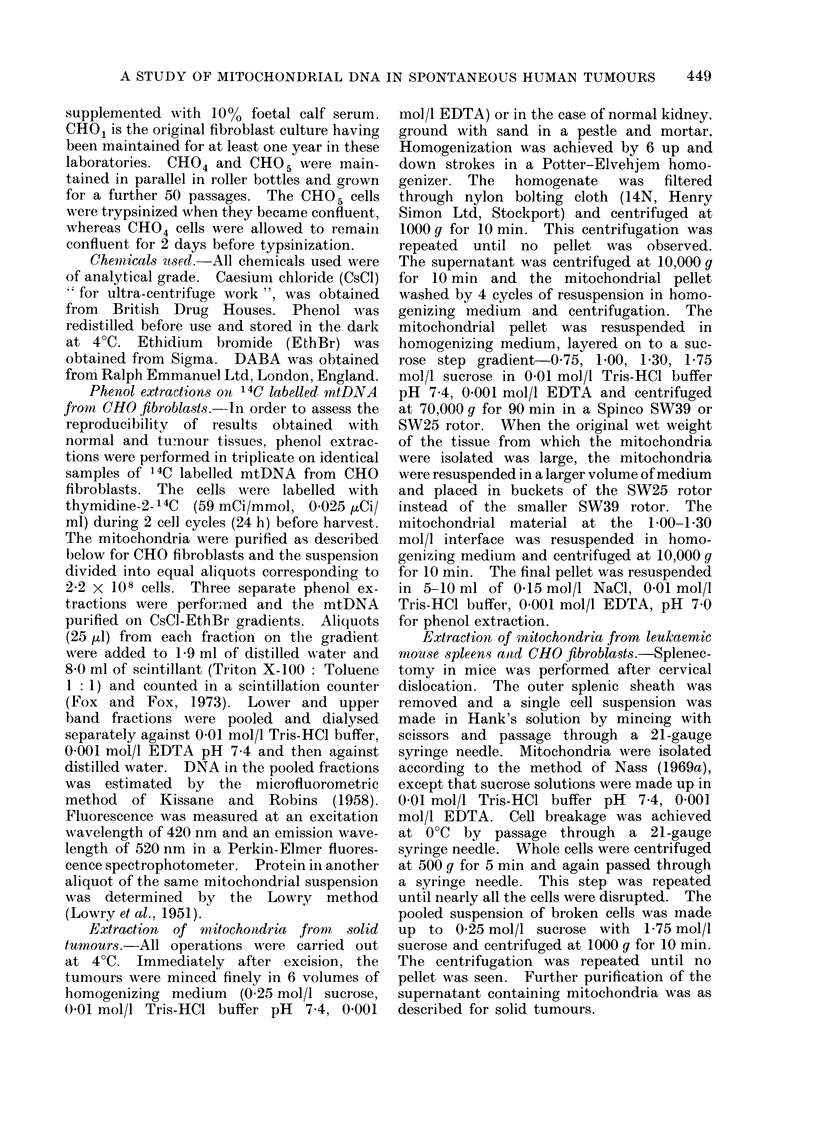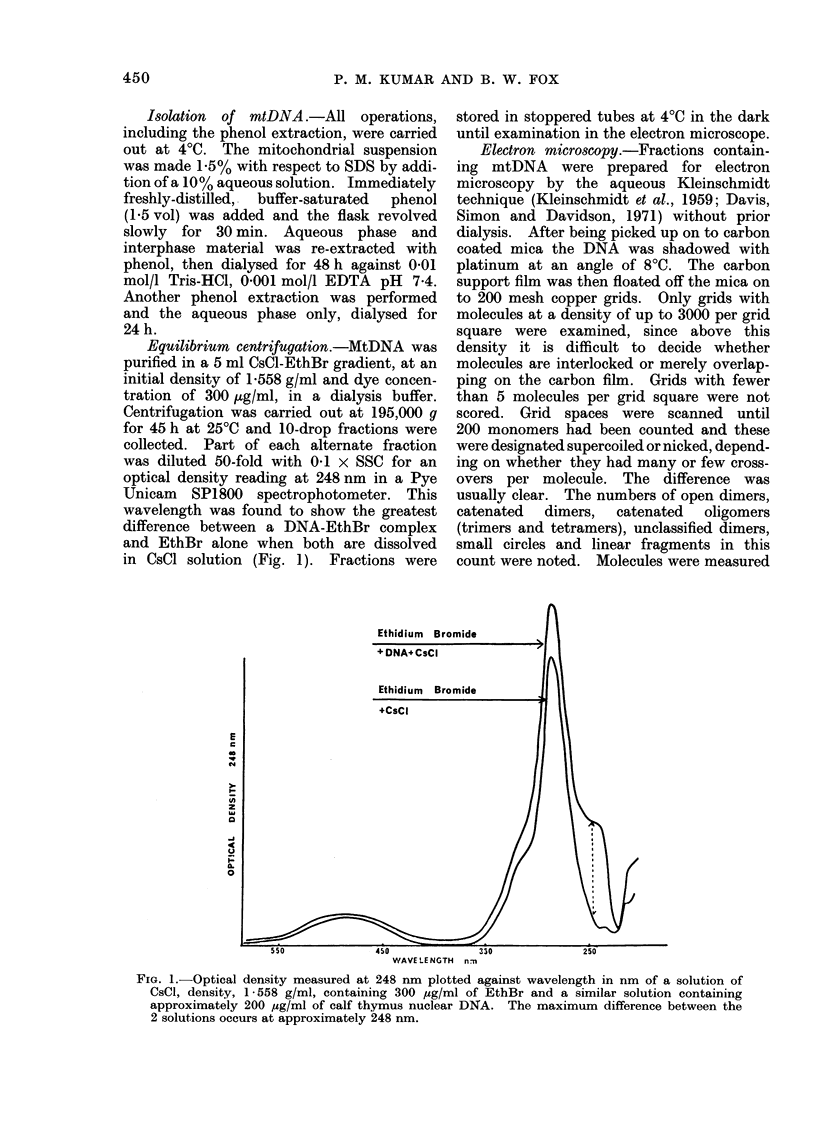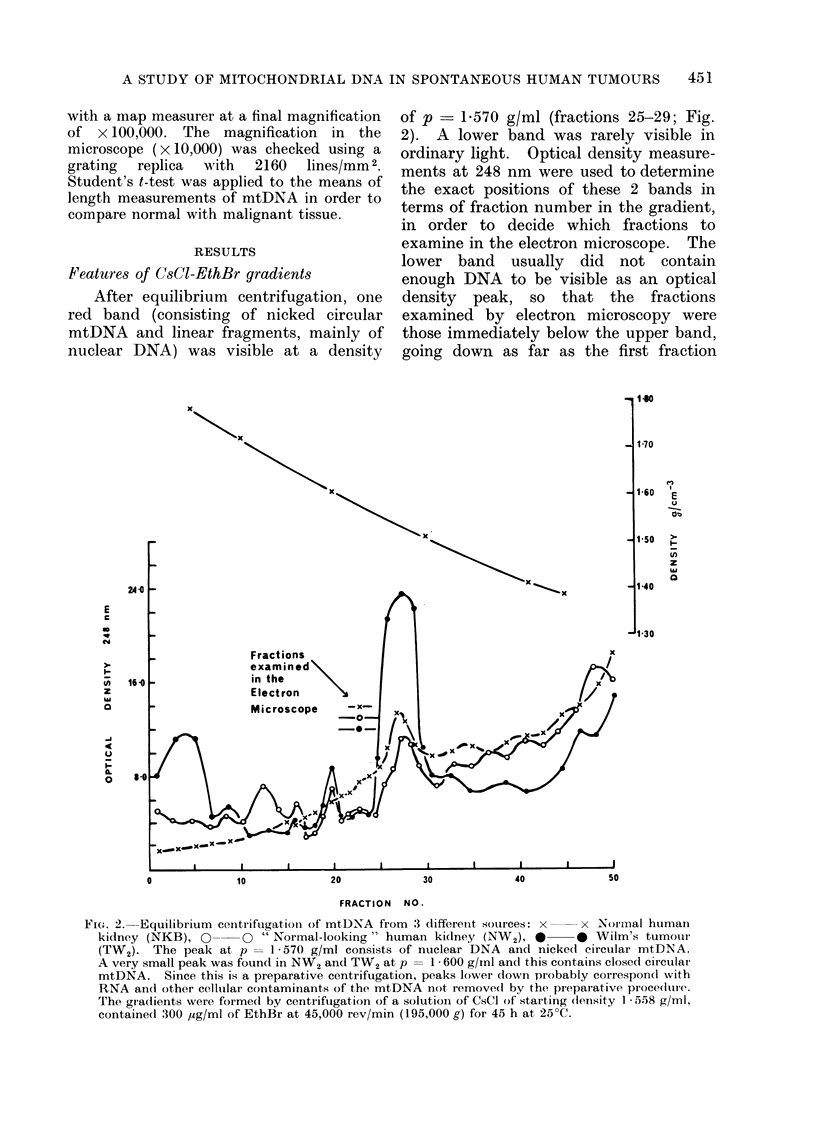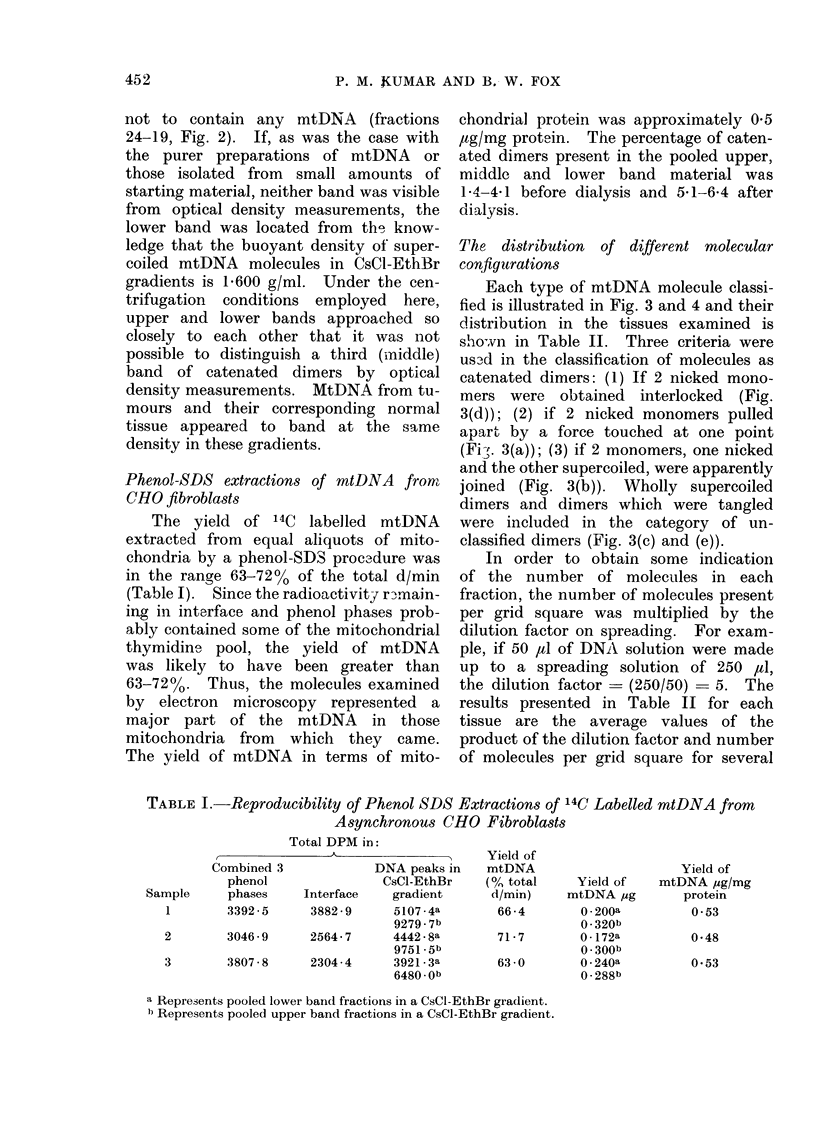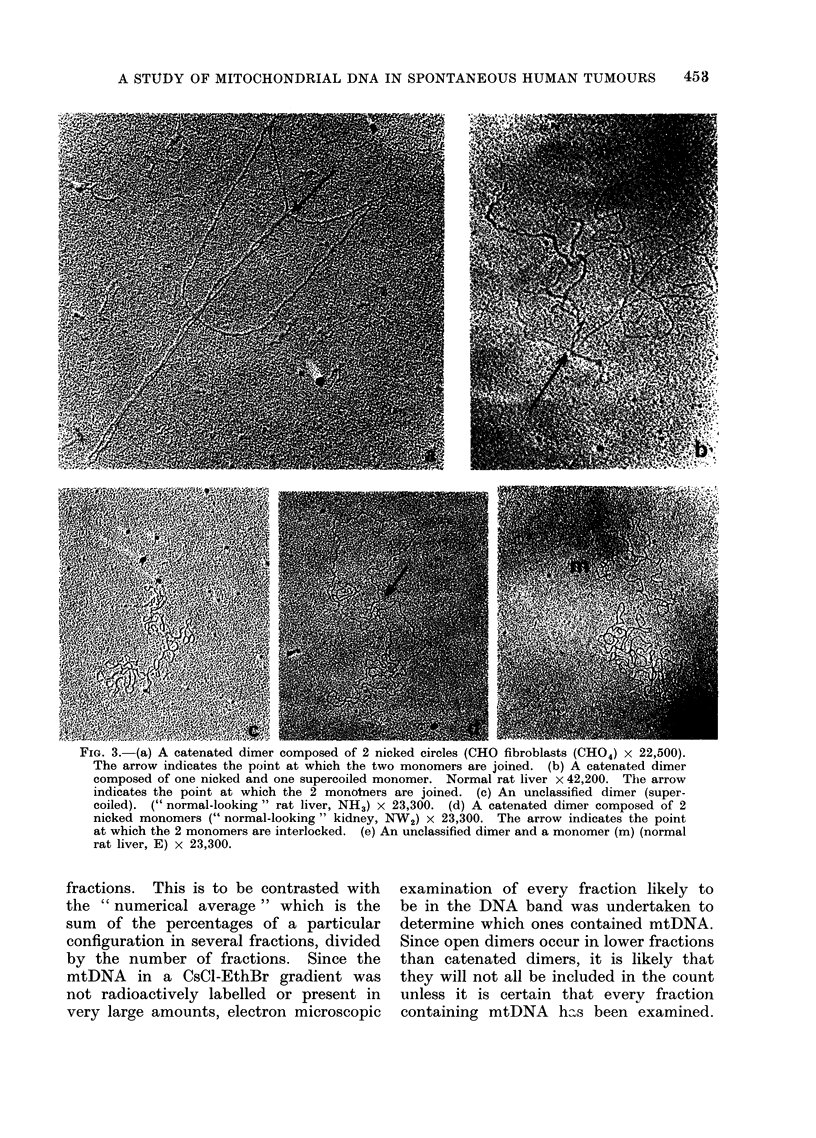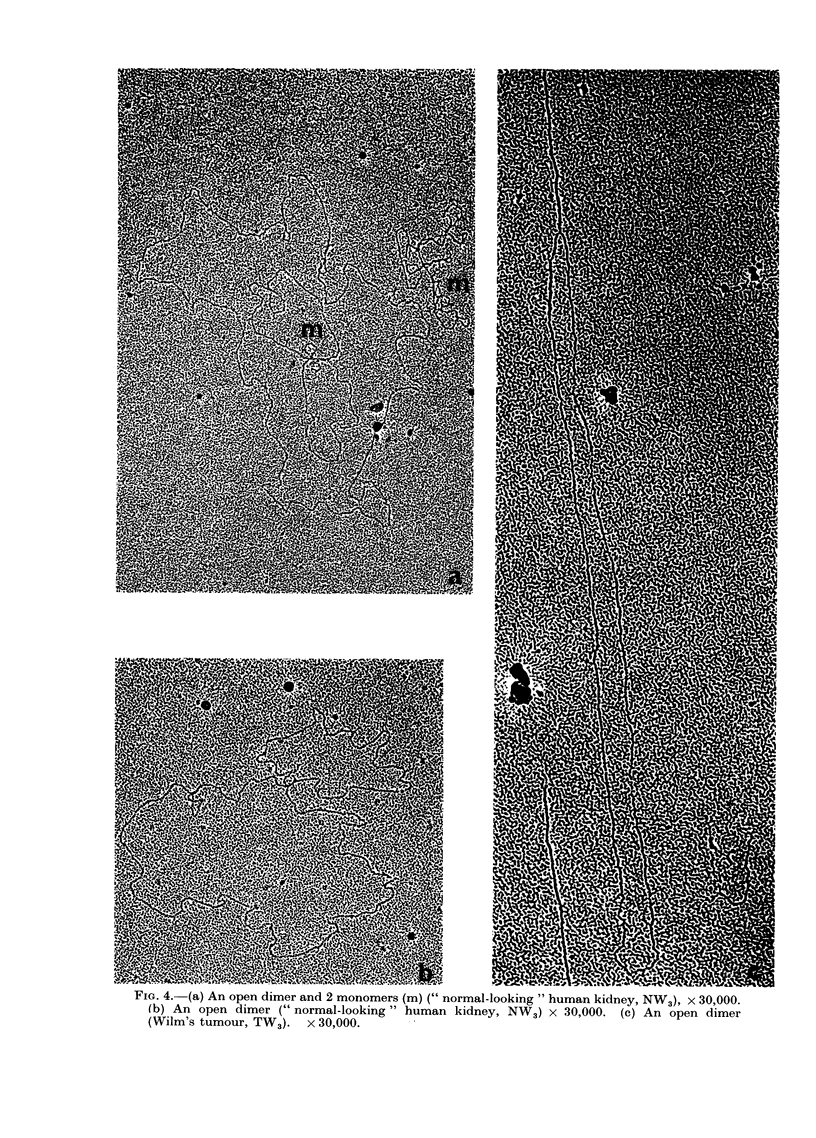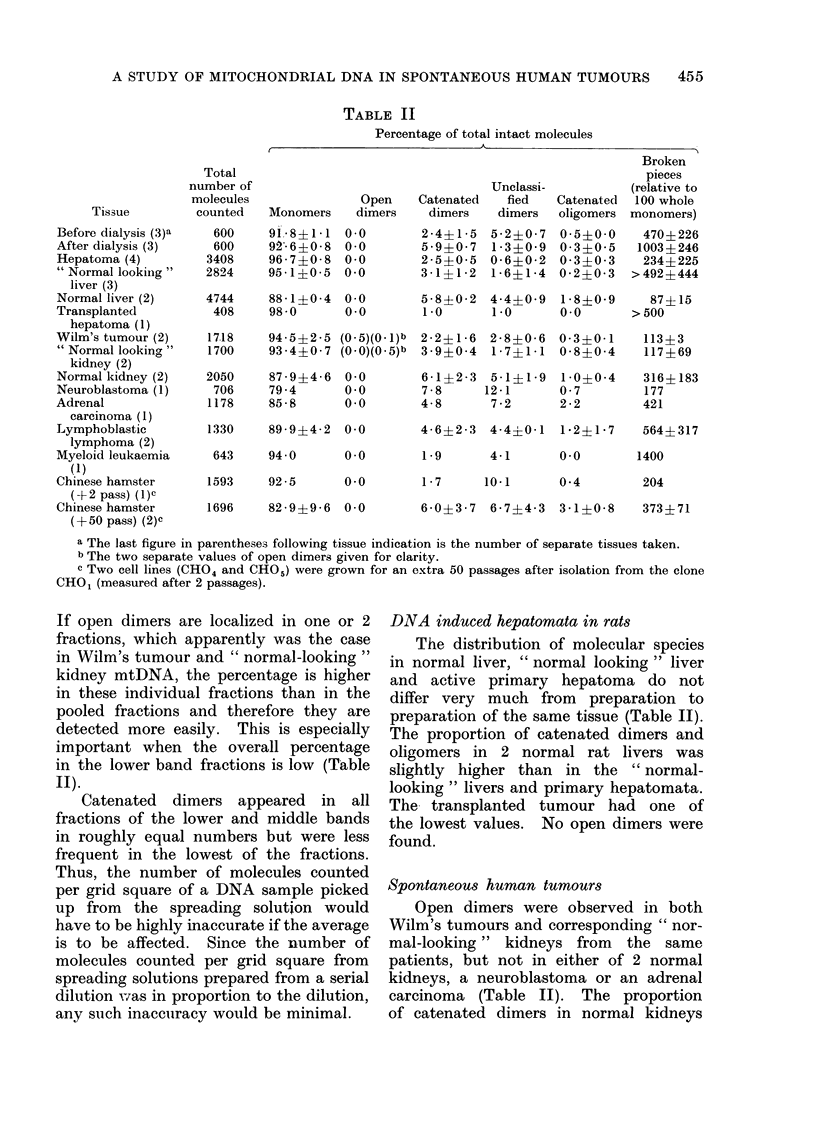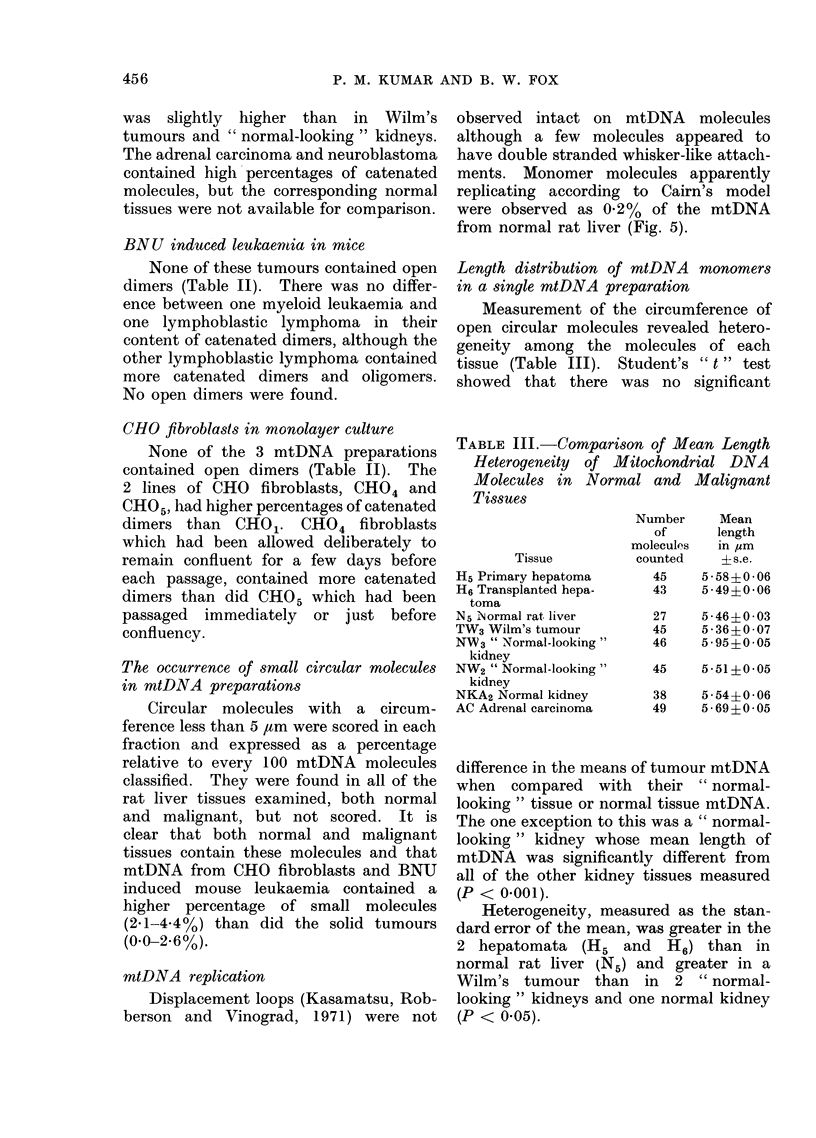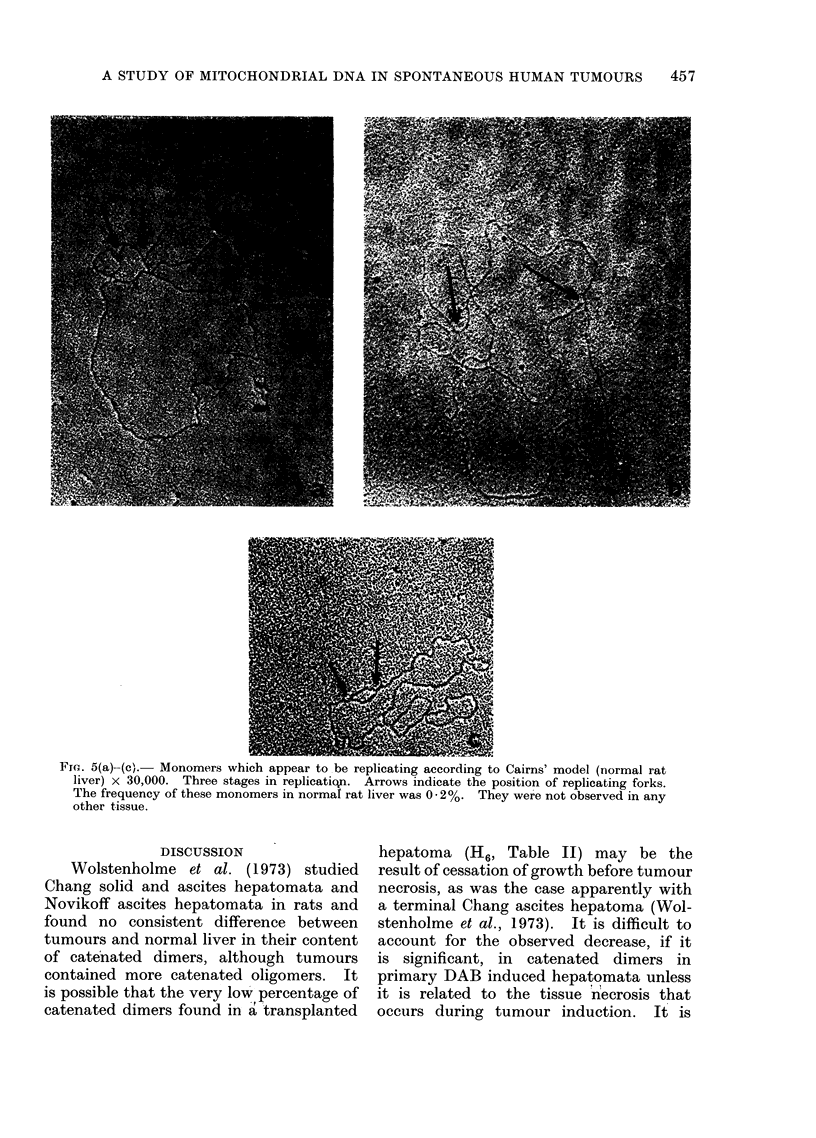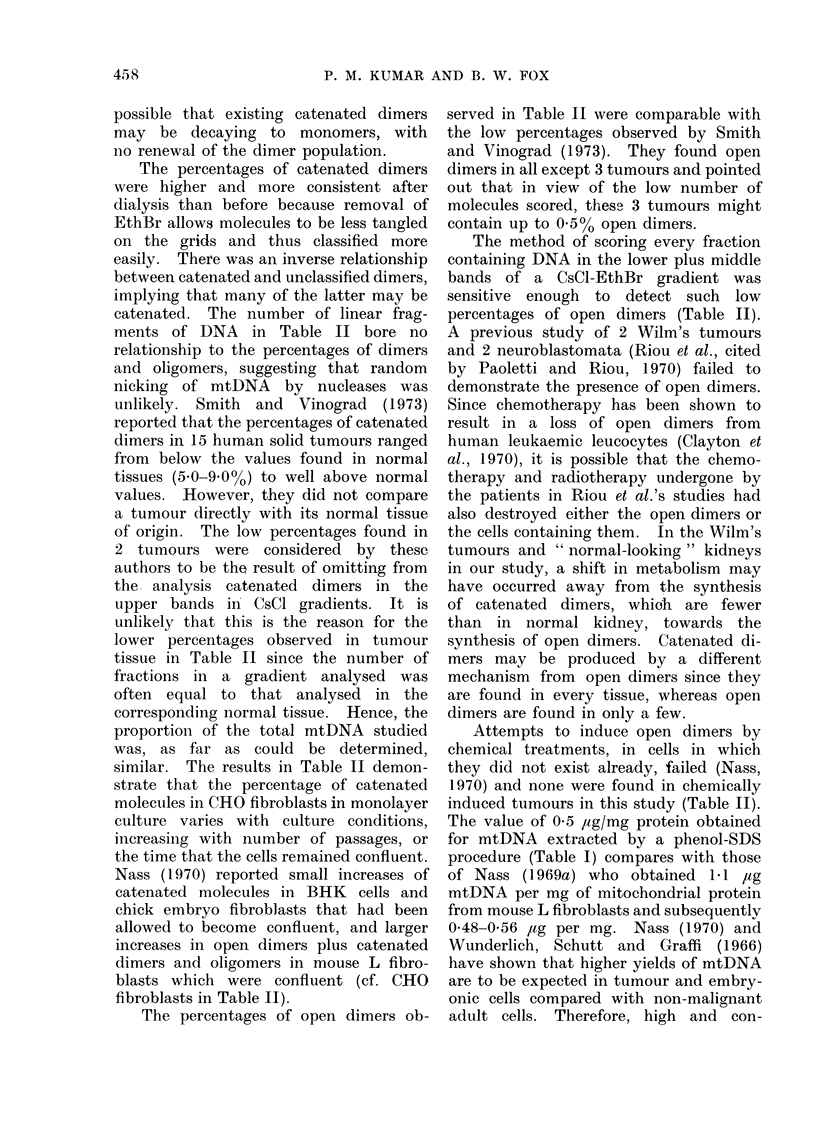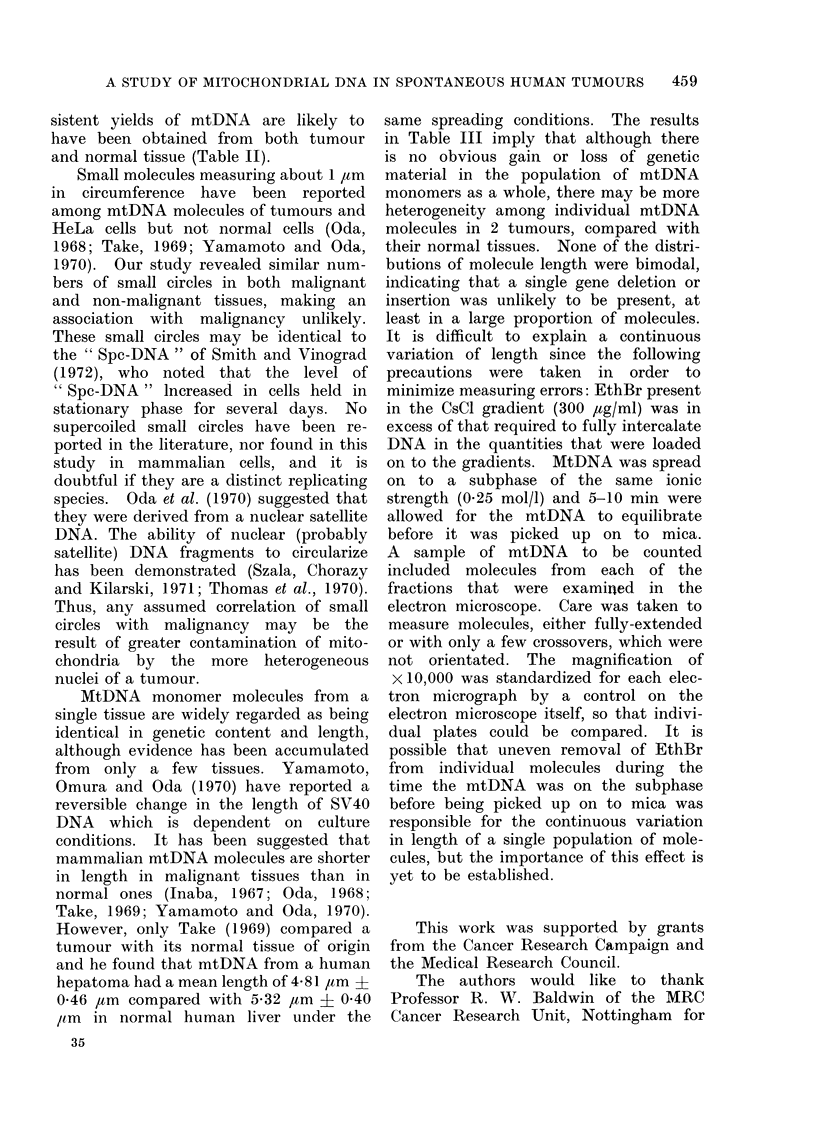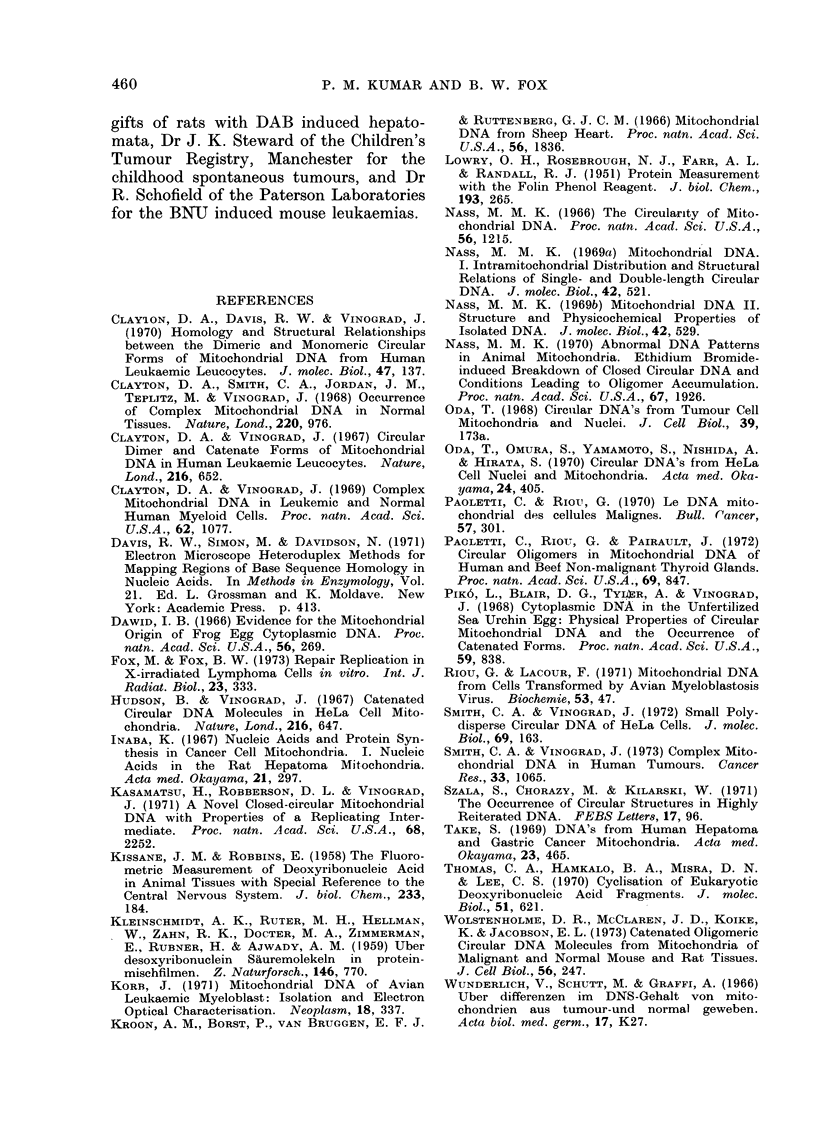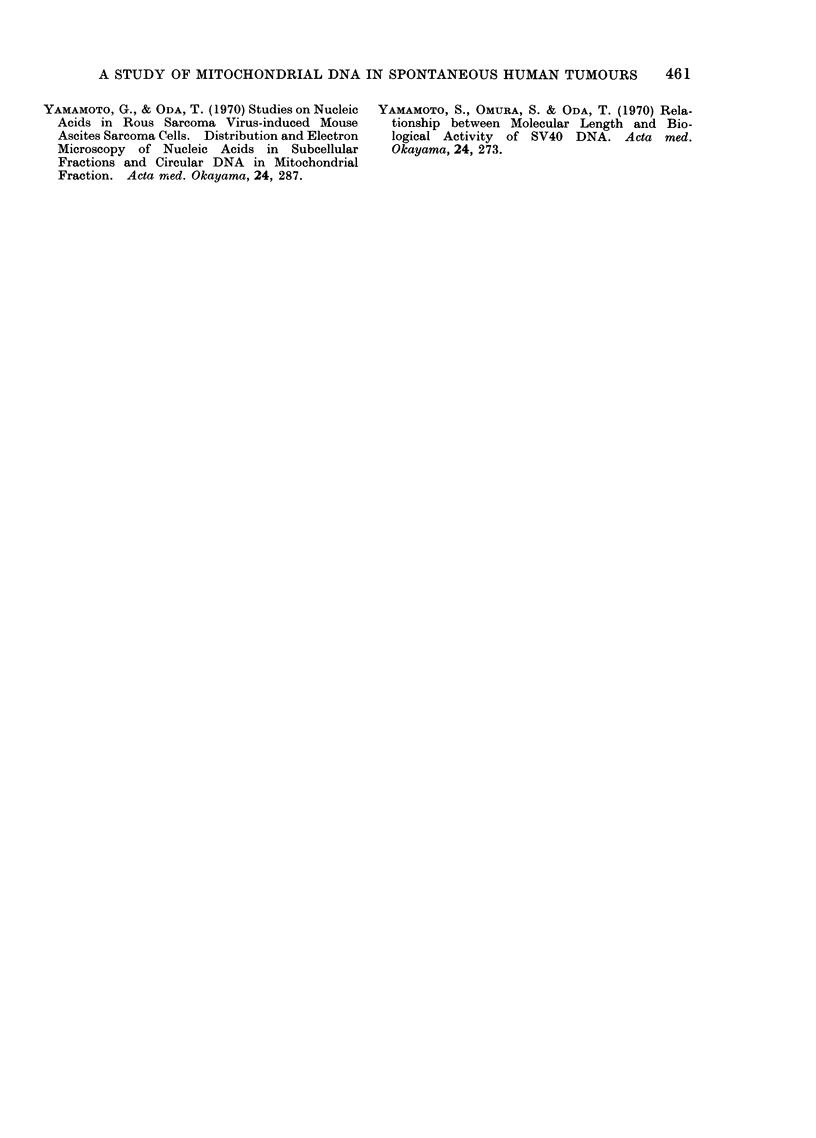# An Electron Microscope Study of Mitochondrial DNA in Spontaneous Human Tumours and Chemically Induced Animal Tumours

**DOI:** 10.1038/bjc.1974.97

**Published:** 1974-06

**Authors:** P. M. Kumar, B. W. Fox

## Abstract

**Images:**


Br. J. Cancer (1974) 29, 447

### AN ELECTRON MICROSCOPE STUDY OF MITOCHONDRIAL DNA IN SPONTANEOUS HUMAN TUMOURS AND CHEMICALLY INDUCED ANIMAL TUMOURS

P. M. KUMAR AND B. W. FOX

*From the Paterson Laboratories, Christie Hospital and Holt Radium Institute, Manchester M20 9BX, U.K.*

Received 20 February 1974. Accepted 12 March 1974

**Summary.—MtDNA was extracted by a phenol method from transplanted and primary DAB induced hepatomata in male Wistar rats, normal rat liver, spontaneous human tumours (2 Wilm's tumours, one neuroblastoma and one adrenal carcinoma), as well as 2 specimens of normal human kidney, BNU induced " leukaemias " in mice and CHO fibroblasts in monolayer culture. The proportion of monomers, catenated dimers and oligomers, open dimers and small circles was determined by electron microscopy of the fractions comprising lower and middle DNA bands in a CsCl-EthBr gradient. Tumours were compared where possible with their normal tissue of origin. Open dimers were found in 2 Wilm's tumours and their attached " normal-looking " kidney tissue but not in normal, non-malignant kidney or any other tissue studied. In Wilm's tumours, the occurrence of open dimers is far from being an all-or-none phenomenon. Malignancy produced little change in the relative proportions of catenated dimers and oligomers in the tissues studied. Small circles were found associated with mtDNA from every tissue. Tumour mtDNA was not more heterogeneous in length than monomers from the corresponding normal tissue, neither was the mean length of tumour mtDNA significantly different from its corresponding normal mtDNA.**

MtDNA exists, with little variation, as circular molecules approximately 5 $\mu$m in circumference, in animals throughout the evolutionary scale from nematodes to man. Departures from the usual 5$\mu$m monomer size occur as interlocked molecules (catenated dimers and oligomers), double length molecules (open dimers) and very small molecules, less than 5 $\mu$m in circumference, which may not be mitochondrial in origin (Smith and Vinograd, 1972). Catenated dimers have been reported in many tissues (Dawid, 1966; Kroon *et al.*, 1966; Nass, 1966; Hudson and Vinograd, 1967; Clayton *et al.*, 1968; Pikó *et al.*, 1968), and are now considered a feature of all normal tissues in mammals. It is unlikely that an elevated proportion of catenated dimers in mtDNA is a feature of malignancy (Paoletti and Riou, 1970), as was hitherto supposed.

At the commencement of this study, no open dimers had been found in a large number of normal mammalian tissues examined by Clayton *et al.* (1968), although human placenta had yielded one open dimer in a count of 3000 molecules. They were found to make up to 40% of molecules in peripheral blood cells in human granulocytic leukaemia (Clayton and Vinograd, 1967, 1969; Clayton, Davis and Vinograd, 1970) and the fact that neither bone marrow tissue nor blood cells from patients with either a myeloid metaplasia or a leukemoid reaction contained open dimers implied that they were a feature of malignancy in white blood cells. Korb (1971) described open dimers in avian myeloblastosis virus induced leukaemia in chickens, to a level of 16% in the middle band of a CsCl-EthBr gradient. Riou and Lacour (1971), in a study of the same leukaemia, detected only 3 open dimers among more than 1900 molecules classified by them. Open dimers had been described in 11 of 14 solid tumours (Smith and Vinograd, cited by Paoletti and Riou, 1970) but in none of 4 neuroblastomata and Wilm's tumours that had undergone chemotherapy and irradiation (Riou *et al.*, cited by Paoletti and Riou, 1970). Nass (1969*b*) reported them in mouse L fibroblasts, but had not tested the malignancy of the cells *in vivo*, and they were also observed in hamster tumours induced *in vivo* by adenoviruses 7 and 12, but only after the cells had been passaged many times in tissue culture (Riou and Delain, cited by Paoletti and Riou, 1970). Since the commencement of this study, open dimers have been found to make up a large proportion of mtDNA in normal human and bovine thyroid gland (Paoletti, Riou and Pairault, 1972).

In view of the limited evidence linking the occurrence of open dimers with malignancy, it was considered worthwhile to investigate the mtDNA of other spontaneous human solid tumours for their content of open dimers and catenated dimers, with appropriate normal human tissues as controls where possible. BNU induced mouse leukaemia and 4-DAB induced rat hepatomata were also examined since no investigation into the mtDNA of chemically induced tumours had been reported.

The following abbreviations are used throughout this paper: mtDNA, mitochondrial DNA; CsCl, caesium chloride; EthBr, ethidium bromide; DAB, dimethylaminoazobenzene; BNU, butylnitrosourea; CHO, Chinese hamster ovary; SSC, saline-sodium citrate; SDS, sodium-dodecyl sulphate; BHK, baby hamster kidney; DABA, 3,5-diaminobenzoic acid.

### MATERIALS AND METHODS

*Primary hepatomata.*—Primary hepatomata were induced in male Wistar rats (Nottingham strain) by continuous feeding of 0·06% 4-DAB in the diet for several weeks. This diet was then replaced by a normal Oxoid diet and the animals sacrificed 9–12 weeks later. In 3 animals, only the right lobes of the liver had a grossly altered appearance to the naked eye. The livers of these animals were divided into the active tumour ($H_3$, $H_4$, $H_5$) and " normal-looking " liver ($NH_3$, $NH_4$, $NH_5$). Active tumour and " normal-looking " liver were processed separately at the same time as a normal rat liver.

*Transplanted DAB induced hepatomata.*—A transplanted hepatoma ($H_6$) was obtained after 30 serial subcutaneous transplants of solid tumour tissue in Wistar rats. The animal was sacrificed 13 days after its transplant, before the tumour began to be necrotic.

*Human tumours.*—Four human tumours comprising 2 Wilm's tumours, one neuroblastoma and one adrenal carcinoma were obtained after surgical removal from children. None had been irradiated or given chemotherapy before surgery. In the case of the Wilm's tumours, part of each kidney was grossly normal in appearance ($NW_2$, $NW_3$) and was separated from the active tumour ($TW_2$, $TW_3$). A kidney was removed from a patient with a neuroblastoma (NKB) and also from a patient with an adrenal carcinoma ($NKA_2$) since the tumours were adhering to the kidneys and infiltration was suspected. These 2 kidneys were found on histological examination not to contain any tumour tissue and were therefore used as normal controls for the Wilm's tumours.

*BNU induced " leukaemia ".*—A lymphoblastic lymphoma with enlarged spleen was induced in mice by continuous feeding of 200 mg/l of BNU in the drinking water for several months until a tumour was induced. The lymphoma so induced was maintained by serial passage *in vivo* every 10 days, using an intraperitoneal injection of $5 \times 10^5$ leukaemic cells obtained from a spleen suspension. In mice from which the thymus had been removed before feeding BNU, a myeloid type of leukaemia developed and the spleen was larger. The intention was to compare the distribution of open dimers and catenated dimers in the 2 forms of leukaemia. A heterogeneous white blood cell population ($5 \times 10^8$) was obtained from each of 6–10 spleens which were pooled and the mtDNA isolated.

*CHO fibroblasts.*—CHO fibroblasts were grown as monolayers in McCoy's medium supplemented with 10% foetal calf serum. $CHO_1$ is the original fibroblast culture having been maintained for at least one year in these laboratories. $CHO_4$ and $CHO_5$ were maintained in parallel in roller bottles and grown for a further 50 passages. The $CHO_5$ cells were trypsinized when they became confluent, whereas $CHO_4$ cells were allowed to remain confluent for 2 days before typsinization.

*Chemicals used.*—All chemicals used were of analytical grade. Caesium chloride (CsCl) "for ultra-centrifuge work", was obtained from British Drug Houses. Phenol was redistilled before use and stored in the dark at 4°C. Ethidium bromide (EthBr) was obtained from Sigma. DABA was obtained from Ralph Emmanuel Ltd, London, England.

*Phenol extractions on $^{14}C$ labelled mtDNA from CHO fibroblasts.*—In order to assess the reproducibility of results obtained with normal and tumour tissues, phenol extractions were performed in triplicate on identical samples of $^{14}C$ labelled mtDNA from CHO fibroblasts. The cells were labelled with thymidine-2-$^{14}C$ (59 mCi/mmol, 0·025 $\mu$Ci/ml) during 2 cell cycles (24 h) before harvest. The mitochondria were purified as described below for CHO fibroblasts and the suspension divided into equal aliquots corresponding to $2 \cdot 2 \times 10^8$ cells. Three separate phenol extractions were performed and the mtDNA purified on CsCl-EthBr gradients. Aliquots (25 $\mu$l) from each fraction on the gradient were added to 1·9 ml of distilled water and 8·0 ml of scintillant (Triton X-100 : Toluene 1 : 1) and counted in a scintillation counter (Fox and Fox, 1973). Lower and upper band fractions were pooled and dialysed separately against 0·01 mol/l Tris-HCl buffer, 0·001 mol/l EDTA pH 7·4 and then against distilled water. DNA in the pooled fractions was estimated by the microfluorometric method of Kissane and Robins (1958). Fluorescence was measured at an excitation wavelength of 420 nm and an emission wavelength of 520 nm in a Perkin-Elmer fluorescence spectrophotometer. Protein in another aliquot of the same mitochondrial suspension was determined by the Lowry method (Lowry *et al.*, 1951).

*Extraction of mitochondria from solid tumours.*—All operations were carried out at 4°C. Immediately after excision, the tumours were minced finely in 6 volumes of homogenizing medium (0·25 mol/l sucrose, 0·01 mol/l Tris-HCl buffer pH 7·4, 0·001 mol/l EDTA) or in the case of normal kidney. ground with sand in a pestle and mortar. Homogenization was achieved by 6 up and down strokes in a Potter–Elvehjem homogenizer. The homogenate was filtered through nylon bolting cloth (14N, Henry Simon Ltd, Stockport) and centrifuged at 1000 *g* for 10 min. This centrifugation was repeated until no pellet was observed. The supernatant was centrifuged at 10,000 *g* for 10 min and the mitochondrial pellet washed by 4 cycles of resuspension in homogenizing medium and centrifugation. The mitochondrial pellet was resuspended in homogenizing medium, layered on to a sucrose step gradient—0·75, 1·00, 1·30, 1·75 mol/l sucrose in 0·01 mol/l Tris-HCl buffer pH 7·4, 0·001 mol/l EDTA and centrifuged at 70,000 *g* for 90 min in a Spinco SW39 or SW25 rotor. When the original wet weight of the tissue from which the mitochondria were isolated was large, the mitochondria were resuspended in a larger volume of medium and placed in buckets of the SW25 rotor instead of the smaller SW39 rotor. The mitochondrial material at the 1·00–1·30 mol/l interface was resuspended in homogenizing medium and centrifuged at 10,000 *g* for 10 min. The final pellet was resuspended in 5–10 ml of 0·15 mol/l NaCl, 0·01 mol/l Tris-HCl buffer, 0·001 mol/l EDTA, pH 7·0 for phenol extraction.

*Extraction of mitochondria from leukaemic mouse spleens and CHO fibroblasts.*—Splenectomy in mice was performed after cervical dislocation. The outer splenic sheath was removed and a single cell suspension was made in Hank's solution by mincing with scissors and passage through a 21-gauge syringe needle. Mitochondria were isolated according to the method of Nass (1969*a*), except that sucrose solutions were made up in 0·01 mol/l Tris-HCl buffer pH 7·4, 0·001 mol/l EDTA. Cell breakage was achieved at 0°C by passage through a 21-gauge syringe needle. Whole cells were centrifuged at 500 *g* for 5 min and again passed through a syringe needle. This step was repeated until nearly all the cells were disrupted. The pooled suspension of broken cells was made up to 0·25 mol/l sucrose with 1·75 mol/l sucrose and centrifuged at 1000 *g* for 10 min. The centrifugation was repeated until no pellet was seen. Further purification of the supernatant containing mitochondria was as described for solid tumours.

*Isolation of mtDNA.*—All operations, including the phenol extraction, were carried out at 4°C. The mitochondrial suspension was made 1·5% with respect to SDS by addition of a 10% aqueous solution. Immediately freshly-distilled, buffer-saturated phenol (1·5 vol) was added and the flask revolved slowly for 30 min. Aqueous phase and interphase material was re-extracted with phenol, then dialysed for 48 h against 0·01 mol/l Tris-HCl, 0·001 mol/l EDTA pH 7·4. Another phenol extraction was performed and the aqueous phase only, dialysed for 24 h.

*Equilibrium centrifugation.*—MtDNA was purified in a 5 ml CsCl-EthBr gradient, at an initial density of 1·558 g/ml and dye concentration of 300 μg/ml, in a dialysis buffer. Centrifugation was carried out at 195,000 *g* for 45 h at 25°C and 10-drop fractions were collected. Part of each alternate fraction was diluted 50-fold with 0·1 × SSC for an optical density reading at 248 nm in a Pye Unicam SP1800 spectrophotometer. This wavelength was found to show the greatest difference between a DNA-EthBr complex and EthBr alone when both are dissolved in CsCl solution (Fig. 1). Fractions were stored in stoppered tubes at 4°C in the dark until examination in the electron microscope.

*Electron microscopy.*—Fractions containing mtDNA were prepared for electron microscopy by the aqueous Kleinschmidt technique (Kleinschmidt *et al.*, 1959; Davis, Simon and Davidson, 1971) without prior dialysis. After being picked up on to carbon coated mica the DNA was shadowed with platinum at an angle of 8°C. The carbon support film was then floated off the mica on to 200 mesh copper grids. Only grids with molecules at a density of up to 3000 per grid square were examined, since above this density it is difficult to decide whether molecules are interlocked or merely overlapping on the carbon film. Grids with fewer than 5 molecules per grid square were not scored. Grid spaces were scanned until 200 monomers had been counted and these were designated supercoiled or nicked, depending on whether they had many or few crossovers per molecule. The difference was usually clear. The numbers of open dimers, catenated dimers, catenated oligomers (trimers and tetramers), unclassified dimers, small circles and linear fragments in this count were noted. Molecules were measured

FIG. 1.—Optical density measured at 248 nm plotted against wavelength in nm of a solution of CsCl, density, 1·558 g/ml, containing 300 μg/ml of EthBr and a similar solution containing approximately 200 μg/ml of calf thymus nuclear DNA. The maximum difference between the 2 solutions occurs at approximately 248 nm.

with a map measurer at a final magnification of ×100,000. The magnification in the microscope (×10,000) was checked using a grating replica with 2160 lines/mm[2]. Student's $t$-test was applied to the means of length measurements of mtDNA in order to compare normal with malignant tissue.

### RESULTS

### *Features of CsCl-EthBr gradients*

After equilibrium centrifugation, one red band (consisting of nicked circular mtDNA and linear fragments, mainly of nuclear DNA) was visible at a density of $p = 1{\cdot}570$ g/ml (fractions 25–29; Fig. 2). A lower band was rarely visible in ordinary light. Optical density measurements at 248 nm were used to determine the exact positions of these 2 bands in terms of fraction number in the gradient, in order to decide which fractions to examine in the electron microscope. The lower band usually did not contain enough DNA to be visible as an optical density peak, so that the fractions examined by electron microscopy were those immediately below the upper band, going down as far as the first fraction

FIG. 2.—Equilibrium centrifugation of mtDNA from 3 different sources: ×——× Normal human kidney (NKB), ○——○ " Normal-looking " human kidney ($NW_2$), ●——● Wilm's tumour ($TW_2$). The peak at $p = 1 \cdot 570$ g/ml consists of nuclear DNA and nicked circular mtDNA. A very small peak was found in $NW_2$ and $TW_2$ at $p = 1 \cdot 600$ g/ml and this contains closed circular mtDNA. Since this is a preparative centrifugation, peaks lower down probably correspond with RNA and other cellular contaminants of the mtDNA not removed by the preparative procedure. The gradients were formed by centrifugation of a solution of CsCl of starting density 1·558 g/ml, contained 300 μg/ml of EthBr at 45,000 rev/min (195,000 $g$) for 45 h at 25°C.

not to contain any mtDNA (fractions 24–19, Fig. 2). If, as was the case with the purer preparations of mtDNA or those isolated from small amounts of starting material, neither band was visible from optical density measurements, the lower band was located from the knowledge that the buoyant density of supercoiled mtDNA molecules in CsCl-EthBr gradients is 1·600 g/ml. Under the centrifugation conditions employed here, upper and lower bands approached so closely to each other that it was not possible to distinguish a third (middle) band of catenated dimers by optical density measurements. MtDNA from tumours and their corresponding normal tissue appeared to band at the same density in these gradients.

*Phenol-SDS extractions of mtDNA from CHO fibroblasts*

The yield of $^{14}C$ labelled mtDNA extracted from equal aliquots of mitochondria by a phenol-SDS procedure was in the range 63–72% of the total d/min (Table I). Since the radioactivity remaining in interface and phenol phases probably contained some of the mitochondrial thymidine pool, the yield of mtDNA was likely to have been greater than 63–72%. Thus, the molecules examined by electron microscopy represented a major part of the mtDNA in those mitochondria from which they came. The yield of mtDNA in terms of mitochondrial protein was approximately 0·5 $\mu$g/mg protein. The percentage of catenated dimers present in the pooled upper, middle and lower band material was 1·4–4·1 before dialysis and 5·1–6·4 after dialysis.

*The distribution of different molecular configurations*

Each type of mtDNA molecule classified is illustrated in Fig. 3 and 4 and their distribution in the tissues examined is shown in Table II. Three criteria were used in the classification of molecules as catenated dimers: (1) If 2 nicked monomers were obtained interlocked (Fig. 3(d)); (2) if 2 nicked monomers pulled apart by a force touched at one point (Fig. 3(a)); (3) if 2 monomers, one nicked and the other supercoiled, were apparently joined (Fig. 3(b)). Wholly supercoiled dimers and dimers which were tangled were included in the category of unclassified dimers (Fig. 3(c) and (e)).

In order to obtain some indication of the number of molecules in each fraction, the number of molecules present per grid square was multiplied by the dilution factor on spreading. For example, if 50 $\mu$l of DNA solution were made up to a spreading solution of 250 $\mu$l, the dilution factor = (250/50) = 5. The results presented in Table II for each tissue are the average values of the product of the dilution factor and number of molecules per grid square for several

TABLE I.—*Reproducibility of Phenol SDS Extractions of $^{14}C$ Labelled mtDNA from Asynchronous CHO Fibroblasts*

| Sample | Total DPM in: Combined 3 phenol phases | Interface | DNA peaks in CsCl-EthBr gradient | Yield of mtDNA (% total d/min) | Yield of mtDNA $\mu$g | Yield of mtDNA $\mu$g/mg protein |
|---|---|---|---|---|---|---|
| 1 | 3392·5 | 3882·9 | 5107·4[a] | 66·4 | 0·200[a] | 0·53 |
| | | | 9279·7[b] | | 0·320[b] | |
| 2 | 3046·9 | 2564·7 | 4442·8[a] | 71·7 | 0·172[a] | 0·48 |
| | | | 9751·5[b] | | 0·300[b] | |
| 3 | 3807·8 | 2304·4 | 3921·3[a] | 63·0 | 0·240[a] | 0·53 |
| | | | 6480·0[b] | | 0·288[b] | |

[a] Represents pooled lower band fractions in a CsCl-EthBr gradient.
[b] Represents pooled upper band fractions in a CsCl-EthBr gradient.

FIG. 3.—(a) A catenated dimer composed of 2 nicked circles (CHO fibroblasts ($CHO_4$) × 22,500). The arrow indicates the point at which the two monomers are joined. (b) A catenated dimer composed of one nicked and one supercoiled monomer. Normal rat liver × 42,200. The arrow indicates the point at which the 2 monomers are joined. (c) An unclassified dimer (supercoiled). (" normal-looking " rat liver, $NH_3$) × 23,300. (d) A catenated dimer composed of 2 nicked monomers (" normal-looking " kidney, $NW_2$) × 23,300. The arrow indicates the point at which the 2 monomers are interlocked. (e) An unclassified dimer and a monomer (m) (normal rat liver, E) × 23,300.

fractions. This is to be contrasted with the " numerical average " which is the sum of the percentages of a particular configuration in several fractions, divided by the number of fractions. Since the mtDNA in a CsCl-EthBr gradient was not radioactively labelled or present in very large amounts, electron microscopic examination of every fraction likely to be in the DNA band was undertaken to determine which ones contained mtDNA. Since open dimers occur in lower fractions than catenated dimers, it is likely that they will not all be included in the count unless it is certain that every fraction containing mtDNA has been examined.

FIG. 4.—(a) An open dimer and 2 monomers (m) (" normal-looking " human kidney, $NW_3$), $\times$ 30,000. (b) An open dimer (" normal-looking " human kidney, $NW_3$) $\times$ 30,000. (c) An open dimer (Wilm's tumour, $TW_3$). $\times$ 30,000.

TABLE II

| Tissue | Total number of molecules counted | Percentage of total intact molecules: Monomers | Open dimers | Catenated dimers | Unclassified dimers | Catenated oligomers | Broken pieces (relative to 100 whole monomers) |
|---|---|---|---|---|---|---|---|
| Before dialysis (3)[a] | 600 | 91·8±1·1 | 0·0 | 2·4±1·5 | 5·2±0·7 | 0·5±0·0 | 470±226 |
| After dialysis (3) | 600 | 92·6±0·8 | 0·0 | 5·9±0·7 | 1·3±0·9 | 0·3±0·5 | 1003±246 |
| Hepatoma (4) | 3408 | 96·7±0·8 | 0·0 | 2·5±0·5 | 0·6±0·2 | 0·3±0·3 | 234±225 |
| " Normal looking " liver (3) | 2824 | 95·1±0·5 | 0·0 | 3·1±1·2 | 1·6±1·4 | 0·2±0·3 | >492±444 |
| Normal liver (2) | 4744 | 88·1±0·4 | 0·0 | 5·8±0·2 | 4·4±0·9 | 1·8±0·9 | 87±15 |
| Transplanted hepatoma (1) | 408 | 98·0 | 0·0 | 1·0 | 1·0 | 0·0 | >500 |
| Wilm's tumour (2) | 1718 | 94·5±2·5 | (0·5)(0·1)[b] | 2·2±1·6 | 2·8±0·6 | 0·3±0·1 | 113±3 |
| " Normal looking " kidney (2) | 1700 | 93·4±0·7 | (0·0)(0·5)[b] | 3·9±0·4 | 1·7±1·1 | 0·8±0·4 | 117±69 |
| Normal kidney (2) | 2050 | 87·9±4·6 | 0·0 | 6·1±2·3 | 5·1±1·9 | 1·0±0·4 | 316±183 |
| Neuroblastoma (1) | 706 | 79·4 | 0·0 | 7·8 | 12·1 | 0·7 | 177 |
| Adrenal carcinoma (1) | 1178 | 85·8 | 0·0 | 4·8 | 7·2 | 2·2 | 421 |
| Lymphoblastic lymphoma (2) | 1330 | 89·9±4·2 | 0·0 | 4·6±2·3 | 4·4±0·1 | 1·2±1·7 | 564±317 |
| Myeloid leukaemia (1) | 643 | 94·0 | 0·0 | 1·9 | 4·1 | 0·0 | 1400 |
| Chinese hamster (+2 pass) (1)[c] | 1593 | 92·5 | 0·0 | 1·7 | 10·1 | 0·4 | 204 |
| Chinese hamster (+50 pass) (2)[c] | 1696 | 82·9±9·6 | 0·0 | 6·0±3·7 | 6·7±4·3 | 3·1±0·8 | 373±71 |

[a] The last figure in parentheses following tissue indication is the number of separate tissues taken.
[b] The two separate values of open dimers given for clarity.
[c] Two cell lines ($CHO_4$ and $CHO_5$) were grown for an extra 50 passages after isolation from the clone $CHO_1$ (measured after 2 passages).

If open dimers are localized in one or 2 fractions, which apparently was the case in Wilm's tumour and " normal-looking " kidney mtDNA, the percentage is higher in these individual fractions than in the pooled fractions and therefore they are detected more easily. This is especially important when the overall percentage in the lower band fractions is low (Table II).

Catenated dimers appeared in all fractions of the lower and middle bands in roughly equal numbers but were less frequent in the lowest of the fractions. Thus, the number of molecules counted per grid square of a DNA sample picked up from the spreading solution would have to be highly inaccurate if the average is to be affected. Since the number of molecules counted per grid square from spreading solutions prepared from a serial dilution was in proportion to the dilution, any such inaccuracy would be minimal.

*DNA induced hepatomata in rats*

The distribution of molecular species in normal liver, " normal looking " liver and active primary hepatoma do not differ very much from preparation to preparation of the same tissue (Table II). The proportion of catenated dimers and oligomers in 2 normal rat livers was slightly higher than in the " normal-looking " livers and primary hepatomata. The transplanted tumour had one of the lowest values. No open dimers were found.

*Spontaneous human tumours*

Open dimers were observed in both Wilm's tumours and corresponding " normal-looking " kidneys from the same patients, but not in either of 2 normal kidneys, a neuroblastoma or an adrenal carcinoma (Table II). The proportion of catenated dimers in normal kidneys was slightly higher than in Wilm's tumours and " normal-looking " kidneys. The adrenal carcinoma and neuroblastoma contained high percentages of catenated molecules, but the corresponding normal tissues were not available for comparison.

*BNU induced leukaemia in mice*

None of these tumours contained open dimers (Table II). There was no difference between one myeloid leukaemia and one lymphoblastic lymphoma in their content of catenated dimers, although the other lymphoblastic lymphoma contained more catenated dimers and oligomers. No open dimers were found.

*CHO fibroblasts in monolayer culture*

None of the 3 mtDNA preparations contained open dimers (Table II). The 2 lines of CHO fibroblasts, $CHO_4$ and $CHO_5$, had higher percentages of catenated dimers than $CHO_1$. $CHO_4$ fibroblasts which had been allowed deliberately to remain confluent for a few days before each passage, contained more catenated dimers than did $CHO_5$ which had been passaged immediately or just before confluency.

*The occurrence of small circular molecules in mtDNA preparations*

Circular molecules with a circumference less than 5 $\mu$m were scored in each fraction and expressed as a percentage relative to every 100 mtDNA molecules classified. They were found in all of the rat liver tissues examined, both normal and malignant, but not scored. It is clear that both normal and malignant tissues contain these molecules and that mtDNA from CHO fibroblasts and BNU induced mouse leukaemia contained a higher percentage of small molecules (2·1–4·4%) than did the solid tumours (0·0–2·6%).

*mtDNA replication*

Displacement loops (Kasamatsu, Robberson and Vinograd, 1971) were not observed intact on mtDNA molecules although a few molecules appeared to have double stranded whisker-like attachments. Monomer molecules apparently replicating according to Cairn's model were observed as 0·2% of the mtDNA from normal rat liver (Fig. 5).

*Length distribution of mtDNA monomers in a single mtDNA preparation*

Measurement of the circumference of open circular molecules revealed heterogeneity among the molecules of each tissue (Table III). Student's " *t* " test showed that there was no significant difference in the means of tumour mtDNA when compared with their " normal-looking " tissue or normal tissue mtDNA. The one exception to this was a " normal-looking " kidney whose mean length of mtDNA was significantly different from all of the other kidney tissues measured ($P < 0{\cdot}001$).

Heterogeneity, measured as the standard error of the mean, was greater in the 2 hepatomata ($H_5$ and $H_6$) than in normal rat liver ($N_5$) and greater in a Wilm's tumour than in 2 " normal-looking " kidneys and one normal kidney ($P < 0{\cdot}05$).

TABLE III.—*Comparison of Mean Length Heterogeneity of Mitochondrial DNA Molecules in Normal and Malignant Tissues*

| Tissue | Number of molecules counted | Mean length in $\mu$m ±s.e. |
|---|---|---|
| $H_5$ Primary hepatoma | 45 | 5·58±0·06 |
| $H_6$ Transplanted hepatoma | 43 | 5·49±0·06 |
| $N_5$ Normal rat liver | 27 | 5·46±0·03 |
| $TW_3$ Wilm's tumour | 45 | 5·36±0·07 |
| $NW_3$ " Normal-looking " kidney | 46 | 5·95±0·05 |
| $NW_2$ " Normal-looking " kidney | 45 | 5·51±0·05 |
| $NKA_2$ Normal kidney | 38 | 5·54±0·06 |
| AC Adrenal carcinoma | 49 | 5·69±0·05 |

FIG. 5(a)–(c).— Monomers which appear to be replicating according to Cairns' model (normal rat liver) × 30,000. Three stages in replication. Arrows indicate the position of replicating forks. The frequency of these monomers in normal rat liver was 0·2%. They were not observed in any other tissue.

### DISCUSSION

Wolstenholme *et al.* (1973) studied Chang solid and ascites hepatomata and Novikoff ascites hepatomata in rats and found no consistent difference between tumours and normal liver in their content of catenated dimers, although tumours contained more catenated oligomers. It is possible that the very low percentage of catenated dimers found in a transplanted hepatoma ($H_6$, Table II) may be the result of cessation of growth before tumour necrosis, as was the case apparently with a terminal Chang ascites hepatoma (Wolstenholme *et al.*, 1973). It is difficult to account for the observed decrease, if it is significant, in catenated dimers in primary DAB induced hepatomata unless it is related to the tissue necrosis that occurs during tumour induction. It is possible that existing catenated dimers may be decaying to monomers, with no renewal of the dimer population.

The percentages of catenated dimers were higher and more consistent after dialysis than before because removal of EthBr allows molecules to be less tangled on the grids and thus classified more easily. There was an inverse relationship between catenated and unclassified dimers, implying that many of the latter may be catenated. The number of linear fragments of DNA in Table II bore no relationship to the percentages of dimers and oligomers, suggesting that random nicking of mtDNA by nucleases was unlikely. Smith and Vinograd (1973) reported that the percentages of catenated dimers in 15 human solid tumours ranged from below the values found in normal tissues (5·0–9·0%) to well above normal values. However, they did not compare a tumour directly with its normal tissue of origin. The low percentages found in 2 tumours were considered by these authors to be the result of omitting from the analysis catenated dimers in the upper bands in CsCl gradients. It is unlikely that this is the reason for the lower percentages observed in tumour tissue in Table II since the number of fractions in a gradient analysed was often equal to that analysed in the corresponding normal tissue. Hence, the proportion of the total mtDNA studied was, as far as could be determined, similar. The results in Table II demonstrate that the percentage of catenated molecules in CHO fibroblasts in monolayer culture varies with culture conditions, increasing with number of passages, or the time that the cells remained confluent. Nass (1970) reported small increases of catenated molecules in BHK cells and chick embryo fibroblasts that had been allowed to become confluent, and larger increases in open dimers plus catenated dimers and oligomers in mouse L fibroblasts which were confluent (cf. CHO fibroblasts in Table II).

The percentages of open dimers observed in Table II were comparable with the low percentages observed by Smith and Vinograd (1973). They found open dimers in all except 3 tumours and pointed out that in view of the low number of molecules scored, these 3 tumours might contain up to 0·5% open dimers.

The method of scoring every fraction containing DNA in the lower plus middle bands of a CsCl-EthBr gradient was sensitive enough to detect such low percentages of open dimers (Table II). A previous study of 2 Wilm's tumours and 2 neuroblastomata (Riou *et al.*, cited by Paoletti and Riou, 1970) failed to demonstrate the presence of open dimers. Since chemotherapy has been shown to result in a loss of open dimers from human leukaemic leucocytes (Clayton *et al.*, 1970), it is possible that the chemotherapy and radiotherapy undergone by the patients in Riou *et al.*'s studies had also destroyed either the open dimers or the cells containing them. In the Wilm's tumours and " normal-looking " kidneys in our study, a shift in metabolism may have occurred away from the synthesis of catenated dimers, which are fewer than in normal kidney, towards the synthesis of open dimers. Catenated dimers may be produced by a different mechanism from open dimers since they are found in every tissue, whereas open dimers are found in only a few.

Attempts to induce open dimers by chemical treatments, in cells in which they did not exist already, failed (Nass, 1970) and none were found in chemically induced tumours in this study (Table II). The value of 0·5 μg/mg protein obtained for mtDNA extracted by a phenol-SDS procedure (Table I) compares with those of Nass (1969*a*) who obtained 1·1 μg mtDNA per mg of mitochondrial protein from mouse L fibroblasts and subsequently 0·48–0·56 μg per mg. Nass (1970) and Wunderlich, Schutt and Graffi (1966) have shown that higher yields of mtDNA are to be expected in tumour and embryonic cells compared with non-malignant adult cells. Therefore, high and consistent yields of mtDNA are likely to have been obtained from both tumour and normal tissue (Table II).

Small molecules measuring about 1 $\mu$m in circumference have been reported among mtDNA molecules of tumours and HeLa cells but not normal cells (Oda, 1968; Take, 1969; Yamamoto and Oda, 1970). Our study revealed similar numbers of small circles in both malignant and non-malignant tissues, making an association with malignancy unlikely. These small circles may be identical to the " Spc-DNA " of Smith and Vinograd (1972), who noted that the level of " Spc-DNA " Increased in cells held in stationary phase for several days. No supercoiled small circles have been reported in the literature, nor found in this study in mammalian cells, and it is doubtful if they are a distinct replicating species. Oda *et al.* (1970) suggested that they were derived from a nuclear satellite DNA. The ability of nuclear (probably satellite) DNA fragments to circularize has been demonstrated (Szala, Chorazy and Kilarski, 1971; Thomas *et al.*, 1970). Thus, any assumed correlation of small circles with malignancy may be the result of greater contamination of mitochondria by the more heterogeneous nuclei of a tumour.

MtDNA monomer molecules from a single tissue are widely regarded as being identical in genetic content and length, although evidence has been accumulated from only a few tissues. Yamamoto, Omura and Oda (1970) have reported a reversible change in the length of SV40 DNA which is dependent on culture conditions. It has been suggested that mammalian mtDNA molecules are shorter in length in malignant tissues than in normal ones (Inaba, 1967; Oda, 1968; Take, 1969; Yamamoto and Oda, 1970). However, only Take (1969) compared a tumour with its normal tissue of origin and he found that mtDNA from a human hepatoma had a mean length of 4·81 $\mu$m $\pm$ 0·46 $\mu$m compared with 5·32 $\mu$m $\pm$ 0·40 $\mu$m in normal human liver under the same spreading conditions. The results in Table III imply that although there is no obvious gain or loss of genetic material in the population of mtDNA monomers as a whole, there may be more heterogeneity among individual mtDNA molecules in 2 tumours, compared with their normal tissues. None of the distributions of molecule length were bimodal, indicating that a single gene deletion or insertion was unlikely to be present, at least in a large proportion of molecules. It is difficult to explain a continuous variation of length since the following precautions were taken in order to minimize measuring errors: EthBr present in the CsCl gradient (300 $\mu$g/ml) was in excess of that required to fully intercalate DNA in the quantities that were loaded on to the gradients. MtDNA was spread on to a subphase of the same ionic strength (0·25 mol/l) and 5–10 min were allowed for the mtDNA to equilibrate before it was picked up on to mica. A sample of mtDNA to be counted included molecules from each of the fractions that were examined in the electron microscope. Care was taken to measure molecules, either fully-extended or with only a few crossovers, which were not orientated. The magnification of $\times$10,000 was standardized for each electron micrograph by a control on the electron microscope itself, so that individual plates could be compared. It is possible that uneven removal of EthBr from individual molecules during the time the mtDNA was on the subphase before being picked up on to mica was responsible for the continuous variation in length of a single population of molecules, but the importance of this effect is yet to be established.

This work was supported by grants from the Cancer Research Campaign and the Medical Research Council.

The authors would like to thank Professor R. W. Baldwin of the MRC Cancer Research Unit, Nottingham for gifts of rats with DAB induced hepatomata, Dr J. K. Steward of the Children's Tumour Registry, Manchester for the childhood spontaneous tumours, and Dr R. Schofield of the Paterson Laboratories for the BNU induced mouse leukaemias.

### REFERENCES

CLAYTON, D. A., DAVIS, R. W. & VINOGRAD, J. (1970) Homology and Structural Relationships between the Dimeric and Monomeric Circular Forms of Mitochondrial DNA from Human Leukaemic Leucocytes. *J. molec. Biol.*, **47**, 137.

CLAYTON, D. A., SMITH, C. A., JORDAN, J. M., TEPLITZ, M. & VINOGRAD, J. (1968) Occurrence of Complex Mitochondrial DNA in Normal Tissues. *Nature, Lond.*, **220**, 976.

CLAYTON, D. A. & VINOGRAD, J. (1967) Circular Dimer and Catenate Forms of Mitochondrial DNA in Human Leukaemic Leucocytes. *Nature, Lond.*, **216**, 652.

CLAYTON, D. A. & VINOGRAD, J. (1969) Complex Mitochondrial DNA in Leukemic and Normal Human Myeloid Cells. *Proc. natn. Acad. Sci. U.S.A.*, **62**, 1077.

DAVIS, R. W., SIMON, M. & DAVIDSON, N. (1971) Electron Microscope Heteroduplex Methods for Mapping Regions of Base Sequence Homology in Nucleic Acids. In *Methods in Enzymology*, Vol. 21. Ed. L. Grossman and K. Moldave. New York: Academic Press. p. 413.

DAWID, I. B. (1966) Evidence for the Mitochondrial Origin of Frog Egg Cytoplasmic DNA. *Proc. natn. Acad. Sci. U.S.A.*, **56**, 269.

FOX, M. & FOX, B. W. (1973) Repair Replication in X-irradiated Lymphoma Cells *in vitro*. *Int. J. Radiat. Biol.*, **23**, 333.

HUDSON, B. & VINOGRAD, J. (1967) Catenated Circular DNA Molecules in HeLa Cell Mitochondria. *Nature, Lond.*, **216**, 647.

INABA, K. (1967) Nucleic Acids and Protein Synthesis in Cancer Cell Mitochondria. I. Nucleic Acids in the Rat Hepatoma Mitochondria. *Acta med. Okayama*, **21**, 297.

KASAMATSU, H., ROBBERSON, D. L. & VINOGRAD, J. (1971) A Novel Closed-circular Mitochondrial DNA with Properties of a Replicating Intermediate. *Proc. natn. Acad. Sci. U.S.A.*, **68**, 2252.

KISSANE, J. M. & ROBBINS, E. (1958) The Fluorometric Measurement of Deoxyribonucleic Acid in Animal Tissues with Special Reference to the Central Nervous System. *J. biol. Chem.*, **233**, 184.

KLEINSCHMIDT, A. K., RUTER, M. H., HELLMAN, W., ZAHN, R. K., DOCTER, M. A., ZIMMERMAN, E., RUBNER, H. & AJWADY, A. M. (1959) Uber desoxyribonuclein Säuremolekeln in proteinmischfilmen. *Z. Naturforsch.*, **146**, 770.

KORB, J. (1971) Mitochondrial DNA of Avian Leukaemic Myeloblast: Isolation and Electron Optical Characterisation. *Neoplasm*, **18**, 337.

KROON, A. M., BORST, P., VAN BRUGGEN, E. F. J. & RUTTENBERG, G. J. C. M. (1966) Mitochondrial DNA from Sheep Heart. *Proc. natn. Acad. Sci. U.S.A.*, **56**, 1836.

LOWRY, O. H., ROSEBROUGH, N. J., FARR, A. L. & RANDALL, R. J. (1951) Protein Measurement with the Folin Phenol Reagent. *J. biol. Chem.*, **193**, 265.

NASS, M. M. K. (1966) The Circularity of Mitochondrial DNA. *Proc. natn. Acad. Sci. U.S.A.*, **56**, 1215.

NASS, M. M. K. (1969*a*) Mitochondrial DNA. I. Intramitochondrial Distribution and Structural Relations of Single- and Double-length Circular DNA. *J. molec. Biol.*, **42**, 521.

NASS, M. M. K. (1969*b*) Mitochondrial DNA II. Structure and Physicochemical Properties of Isolated DNA. *J. molec. Biol.*, **42**, 529.

NASS, M. M. K. (1970) Abnormal DNA Patterns in Animal Mitochondria. Ethidium Bromide-induced Breakdown of Closed Circular DNA and Conditions Leading to Oligomer Accumulation. *Proc. natn. Acad. Sci. U.S.A.*, **67**, 1926.

ODA, T. (1968) Circular DNA's from Tumour Cell Mitochondria and Nuclei. *J. Cell Biol.*, **39**, 173a.

ODA, T., OMURA, S., YAMAMOTO, S., NISHIDA, A. & HIRATA, S. (1970) Circular DNA's from HeLa Cell Nuclei and Mitochondria. *Acta med. Okayama*, **24**, 405.

PAOLETTI, C. & RIOU, G. (1970) Le DNA mitochondrial des cellules Malignes. *Bull. Cancer*, **57**, 301.

PAOLETTI, C., RIOU, G. & PAIRAULT, J. (1972) Circular Oligomers in Mitochondrial DNA of Human and Beef Non-malignant Thyroid Glands. *Proc. natn. Acad. Sci. U.S.A.*, **69**, 847.

PIKÓ, L., BLAIR, D. G., TYLER, A. & VINOGRAD, J. (1968) Cytoplasmic DNA in the Unfertilized Sea Urchin Egg: Physical Properties of Circular Mitochondrial DNA and the Occurrence of Catenated Forms. *Proc. natn. Acad. Sci. U.S.A.*, **59**, 838.

RIOU, G. & LACOUR, F. (1971) Mitochondrial DNA from Cells Transformed by Avian Myeloblastosis Virus. *Biochemie*, **53**, 47.

SMITH, C. A. & VINOGRAD, J. (1972) Small Polydisperse Circular DNA of HeLa Cells. *J. molec. Biol.*, **69**, 163.

SMITH, C. A. & VINOGRAD, J. (1973) Complex Mitochondrial DNA in Human Tumours. *Cancer Res.*, **33**, 1065.

SZALA, S., CHORAZY, M. & KILARSKI, W. (1971) The Occurrence of Circular Structures in Highly Reiterated DNA. *FEBS Letters*, **17**, 96.

TAKE, S. (1969) DNA's from Human Hepatoma and Gastric Cancer Mitochondria. *Acta med. Okayama*, **23**, 465.

THOMAS, C. A., HAMKALO, B. A., MISRA, D. N. & LEE, C. S. (1970) Cyclisation of Eukaryotic Deoxyribonucleic Acid Fragments. *J. molec. Biol.*, **51**, 621.

WOLSTENHOLME, D. R., MCCLAREN, J. D., KOIKE, K. & JACOBSON, E. L. (1973) Catenated Oligomeric Circular DNA Molecules from Mitochondria of Malignant and Normal Mouse and Rat Tissues. *J. Cell Biol.*, **56**, 247.

WUNDERLICH, V., SCHUTT, M. & GRAFFI, A. (1966) Uber differenzen im DNS-Gehalt von mitochondrien aus tumour-und normal geweben. *Acta biol. med. germ.*, **17**, K27.

YAMAMOTO, G., & ODA, T. (1970) Studies on Nucleic Acids in Rous Sarcoma Virus-induced Mouse Ascites Sarcoma Cells. Distribution and Electron Microscopy of Nucleic Acids in Subcellular Fractions and Circular DNA in Mitochondrial Fraction. *Acta med. Okayama*, **24**, 287.

YAMAMOTO, S., OMURA, S. & ODA, T. (1970) Relationship between Molecular Length and Biological Activity of SV40 DNA. *Acta med. Okayama*, **24**, 273.